# A novel KCNQ1 nonsense variant in the isoform-specific first exon causes both jervell and Lange-Nielsen syndrome 1 and long QT syndrome 1: a case report

**DOI:** 10.1186/s12881-017-0430-7

**Published:** 2017-06-08

**Authors:** Motoi Nishimura, Marehiko Ueda, Ryota Ebata, Emi Utsuno, Takuma Ishii, Kazuyuki Matsushita, Osamu Ohara, Naoki Shimojo, Yoshio Kobayashi, Fumio Nomura

**Affiliations:** 1 Division of Clinical Genetics, Chiba University Hospital, 1-8-1 Inohana, Chuo-ku, Chiba City, Chiba Prefecture 260-8670 Japan; 2 Division of Laboratory Medicine, Chiba University Hospital, 1-8-1 Inohana, Chuo-ku, Chiba City, Chiba Prefecture 260-8670 Japan; 3 Department of Cardiovascular Medicine, Chiba University Graduate School of Medicine, 1-8-1 Inohana, Chuo-ku, Chiba City, Chiba Prefecture 260-8670 Japan; 4 Department of Pediatrics, Chiba University Graduate School of Medicine, 1-8-1 Inohana, Chuo-ku, Chiba City, Chiba Prefecture 260-8670 Japan; 5 Kawaguchi Kogyo General Hospital, 1-18-10, Sakae-cho, Kawaguchi, Saitama 332-0017 Japan; 6 Kazusa DNA Reaearch Institute, 2-6-7 Kazusa-kamatari, Kisarazu, Chiba 292-0818 Japan; 7 Divisions of Clinical Mass Spectrometry and Clinical Genetics, Chiba University Hospital, 1-8-1 Inohana, Chuo-ku, Chiba City, Chiba Prefecture 260–8670, Japan

**Keywords:** Case report, Haploinsufficiency, Jervell and Lange-Nielsen Syndrome (JLNS), KCNQ1 (potassium channel Voltage gated, KQT-like subfamily, member 1), Long QT syndrome (LQTS), Romano-Ward syndrome (RWS), Phenotype variety

## Abstract

**Background:**

According to previous KCNQ1 (potassium channel, voltage gated, KQT-like subfamily, member 1) gene screening studies, missense variants, but not nonsense or frame-shift variants, cause the majority of long QT syndrome (LQTS; Romano-Ward syndrome [RWS]) 1 cases. Several missense variants are reported to cause RWS by a dominant-negative mechanism, and some KCNQ1 variants can cause both Jervell and Lange-Nielsen Syndrome (JLNS; in an autosomal recessive manner) and LQTS1 (in an autosomal dominant manner), while other KCNQ1 variants cause only JLNS. The human KCNQ1 gene is known to have two transcript isoforms (kidney isoform and pancreas isoform), and both isoforms can form a functional cardiac potassium channel.

**Case presentation:**

Here, we report a novel nonsense KCNQ1 variant causing not only JLNS, but also significant QTc prolongation identical to RWS in an autosomal dominant manner. Our case study supports that haploinsufficiency in the KCNQ1 gene is causative of significant QTc prolongation identical to RWS. Interestingly, the nonsense variant (NM_000218.2:c.115G > T [p.Glu39X]) locates in exon 1a of KCNQ1, which is a kidney-isoform specific exon. The variant is located closer to the N-terminus than previously identified nonsense or frame-shift variants.

**Conclusion:**

To the best of our knowledge, this is the first report showing that a nonsense variant in exon 1a of KCNQ1, which is the kidney-isoform specific exon, causes JLNS. Our findings may be informative to the genetic pathogenesis of RWS and JLNS caused by KCNQ1 variants.

**Electronic supplementary material:**

The online version of this article (doi:10.1186/s12881-017-0430-7) contains supplementary material, which is available to authorized users.

## Background

**Fig. 1:**
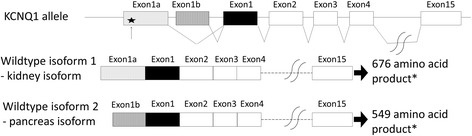
Exon–intron structure and alternative transcripts of the human KCNQ1 gene. The two wild-type isoform transcripts, the kidney and pancreas isoforms, are composed of exons 1a and 1–15 (kidney isoform) encoding 676 amino acids or exons 1b and 1–15 (pancreas isoform) encoding 549 amino acids [1, 4]. The arrow and star indicate the nonsense variant (NM_000218.2:c.115G > T, p.Glu39X) in exon 1a of the KCNQ1 gene. *, both isoforms can form a functional cardiac potassium channel [1, 2]

The human KCNQ1 (potassium channel, voltage gated, KQT-like subfamily, member 1) gene is known to have two transcript isoforms [1]; the longer, kidney isoform encodes a protein of 676 amino acids, while the shorter form is the pancreas isoform (549 amino acids). The kidney KCNQ1 protein is almost identical to pancreatic KCNQ1 protein, except for the highly dissimilar N-terminus region [1]. Both isoforms can form a functional cardiac potassium channel [1, 2]. The kidney isoform contains a specific first exon, known as exon 1a [3, 4] (Fig. 1), which encodes 129 amino acids [4]. The kidney isoform is mainly expressed in the human heart [1], and the shorter isoform (containing exon 1b instead of exon 1a) is also reported to be expressed in the human heart [3]. Thus, both isoforms are expressed at significant levels in the human heart.

Variants in the KCNQ1 gene can cause two hereditary variants of congenital long-QT syndrome (LQTS). One variant is known as long QT syndrome 1 (LQT1) and the other is severe Jervell and Lange-Nielsen syndrome 1 (JLNS1). The typical mode of LQT1 inheritance is autosomal dominant, whereas JLNS1 shows autosomal recessive inheritance or sporadic cases of compound heterozygosity [5].

In LQTS1, the variants may produce different effects in the multimeric cardiac potassium channel. Defective and wild-type protein subunits may coassemble and exert a dominant negative effect on the potassium channel function. Alternatively, some mutant subunits may not coassemble with the wild-type proteins, resulting in a loss of function (haploinsufficiency) [5]. LQTS1 can be caused by loss-of-function variants in the KCNQ1-encoded cardiac potassium channel [6]. Homozygous gene variants in KCNQ1, or compound heterozygous gene variants, may cause the recessive JLNS variant, which is characterized by deafness.

KCNQ1 exhibits different functions in different tissues, which accounts for KCNQ1 variants causing the two syndromes. When KCNQ1 is coupled with the beta-subunit KCNE1, it repolarizes cardiac action potentials [7, 8]. Importantly, transepithelial potassium transport in the inner ear is also associated with the KCNQ1 protein [9], which is why KCNQ1 variants may cause deafness in JLNS1 [8]. Deafness in JLNS is characterized as congenital, bilateral and sensorineural hearing loss [9].

Both frameshift/nonsense variants and missense/splice site variants may cause LQT1 and JLNS1 [10, 11]. Therefore, a precise genotype-phenotype correlation in LQT1 and JLNS1 is not established, which complicates both genetic counseling and clinical risk evaluation in carriers [9, 12–16]. A frameshift variant (NM_000218.2[KCNQ1]:c.567dupG [17]) is reported to cause not only JLNS1 when homozygous, but also causes severe LQT1 when heterozygous. Nevertheless, nonsense and frameshift variants that are generally associated with a non-penetrant phenotype (no symptoms, QTc normal or borderline) have been identified in heterozygous carriers in JLNS1 families [12]. For example, skipping of KCNQ1 exon 1, the first common exon of the kidney and pancreas isoforms (Fig. 1), causes JLNS1 when homozygous, but exhibits an asymptomatic cardiac phenotype with normal QTc interval when heterozygous [4].

Here, we report on a JLNS1 patient with a homozygous nonsense variant in exon 1a of KCNQ1 in a family exhibiting LQT1. Thus, this is the first report that a nonsense variant in the kidney isoform-specific exon (exon 1a) can cause JLNS1.

## Case presentation

**Fig. 2:**
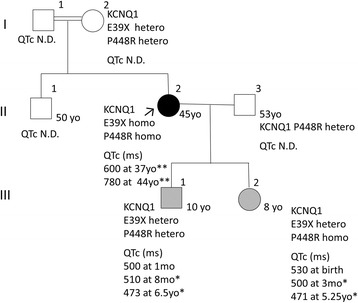
Pedigree of the family. The proband is indicated by a black filled circle and an arrowhead. Squares denote males, and circles denote females. Hatched squares or circles denote significant QTc prolongation. QTc intervals are provided for each individual. *, QTc was measured under beta-blocker treatment (metoprolol). **, QTc was measured under treatment with beta-blockers (atenolol and bisoprolol) and other medication (mexiletine). E39X and P448R, nonsense variant (NM_000218.2:c.115G > T) or common variant (NM_000218.2:c.1343C > G) in the KCNQ1 gene. mo., months old; yo., years old; hetero, heterozygous; homo, homozygous; ms, millisecond; N.D., not determined

The proband (II-2 in Fig. 2) is a 45-year old woman, who first presented to our university hospital at the age of 35 and was referred to us because of her pregnancy. She has congenital deafness, first experienced syncope at the age of 3, and was diagnosed with epilepsy. She was treated with anti-epilepsy medications; however, she subsequently experienced several instances of syncope. At the age of 13, she had a syncope event, and was suspected of having JLNS because of her congenital deafness and prolonged QT interval. Her syncope was diagnosed as an arrhythmic episode when she was aware of tachycardia and as epilepsy when she was not. She also had a subarachnoid hemorrhage at the age of 29.

**Fig. 3:**
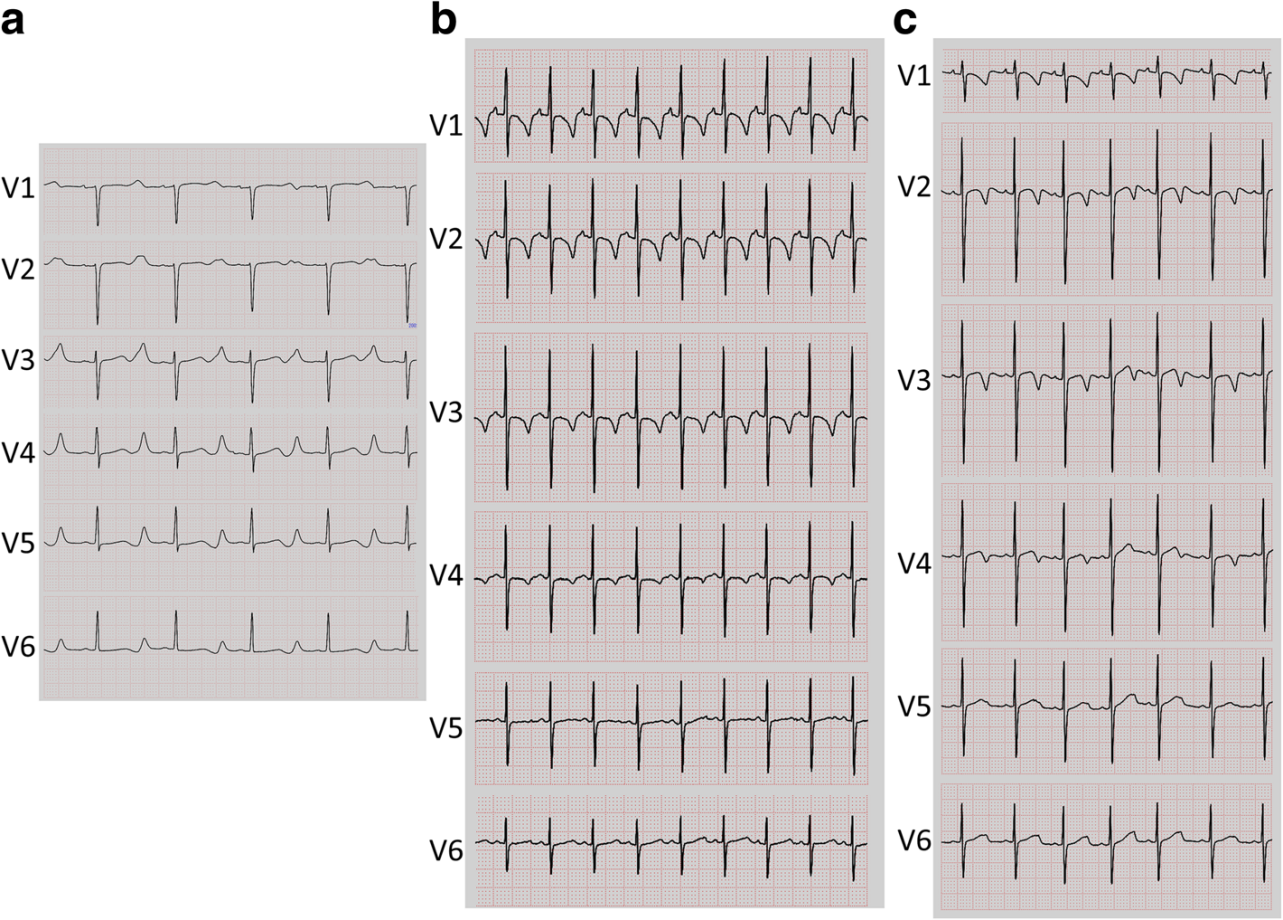
Baseline electrocardiogram (ECG) of the proband (II-2), her son (III-1), and her daughter (III-2). Baseline ECG from the proband at 44 years of age **a**, baseline ECG from the son at 8 months of age **b**, and baseline ECG from the daughter at 5.25 years old **c**. Baseline ECG from the proband **a** was recorded under beta-blockers (atenolol and bisoprolol) and other medication (mexiletine). Baseline ECGs from the children **b**, **c** were recorded under beta-blocker treatment (metoprolol)

When she first presented at our hospital, she was not taking beta-blockers, because of a history of asthma, but was taking mexiletine in addition to phenytoin. Her QTc was found to be prolonged (584 ms) at presentation and administration of atenolol was initiated. She delivered her baby (III-1 in Fig. 2) through Caesarean operation at our hospital at the age of 35. At 37, she delivered her second baby (III-2 in Fig. 2) through Caesarean operation at our hospital. Despite administration of beta-blockers, her QTc remained prolonged (600 msec at the age of 37, 780 msec at 44) (Figs. 2 and 3a), which is not unexpected because treatment with beta-blockers in LQTS1 is not expected to overtly reduce QTc [18]. However, she continued to experience occasional syncope and finally underwent an implantable cardioverter defibrillator (ICD) operation at 38 years of age. Subsequently, she is in a stable clinical condition. Because the proband was suspected of JLNS and both infants had a measured QTc of 500 ms or greater within 1 month after birth, beta blockers were initiated and both children remain in stable condition at ages 10 and 8 (Figs. 2 and 3b, c). QTc of the son (III-1 in Fig. 2) was measured as 500 ms one month after birth, while the QTc of his sister (III-2) was 530 ms at birth.

The father (I-1) and mother (I-2) of the proband were first cousins. There is no history of sudden unexplained syncope or death of children or adults in the immediate family members, despite the prolonged QTc of the children.

### Clinical evaluation of the proband and her family members, and blood collection

Clinical evaluation and consultation of the proband and her family members were performed at Chiba University Hospital. Clinical phenotypes were deduced from the clinical history, physical examinations, and ECG. Blood samples were collected from the proband and her family members following genetic counseling, and written informed consent was obtained prior to sample collection.

### Genetic testing

Genomic DNA was isolated from peripheral blood lymphocytes according to established protocols at our laboratory [19]. Entire coding exons, including the intronic boundaries of the genes, of KCNQ1 (NCBI ref: NM_000218) and other LQT causative genes (KCNH2, SCN5A, KCNE1, KCNE2, KCNJ2, SCN4B, KCNJ5) were amplified by polymerase chain reaction (PCR), according to established protocols in our laboratory. Briefly, 30–100 ng of genomic DNA was subjected to PCR amplification with DNA polymerase (PrimeSTAR GXL DNA Polymerase; Takara Bio Inc., Kusatsu, Japan) and primer sets.

The amplicons were subjected to conventional sequencing with Sanger sequencers (Applied Biosystems 3730/3130 DNA analyzers; Thermo Fisher Scientific, Waltham, MA, USA). The sequence data were processed with Gene Codes Sequencher Software (Takara Bio Inc.) and mapped to the human genome sequence (build GRCh37/hg19).

### Genomic report

Genetic analysis was performed to screen all coding exons and the exon–intron boundaries of the KCNQ1 gene (NCBI ref: NM_000218.2, NP_000209.2) with concurrent screening of other LQT causative genes (KCNH2, SCN5A, KCNE1, KCNE2, KCNJ2, SCN4B, KCNJ5). We detected a novel homozygous nonsense variant, NM_000218.2:c.115G > T (p.Glu39X, in exon 1a), in the KCNQ1 gene of the proband, as well as a homozygous common variant (NM_000218.2:c.1343C > G, p.Pro448Arg) (Additional file 1: Table S1). Genetic screening of her mother (I-2) and children (III-1 and III-2) revealed that they were heterozygous for the nonsense variant (Fig. 2). Her husband (II-3) was also screened and found to be heterozygous for the common variant (NM_000218.2:c.1343C > G, p.Pro448Arg). The proband is a child from a first-cousin marriage, and we have concluded the homozygous nonsense variant in the proband is the cause of her JLNS1. The proband was negative for pathogenic variants in other LQT causative genes, including the KCNE1 gene (Additional file 1: Table S1).

## Discussion and conclusions

To the best of our knowledge, this is the first report showing that a nonsense variant in exon 1a of KCNQ1, which is the kidney-isoform specific exon, causes JLNS. The novel NM_000218.2:c.115G > T (p.Glu39X) variant is located closer to the N-terminus than previously identified pathogenic variants. This nonsense variant can cause not only JLNS, but also significant QTc prolongation that is identical to RWS.

A homozygous common variant (NM_000218.2:c.1343C > G, p.Pro448Arg) was also detected in the KCNQ1 gene of the proband. This common variant is reported to be highly frequent in Asian populations, including the Chinese and Japanese (14 to 28% allele frequency [20–22]), and may have an effect on the channel current [21]. The p.Pro448Arg common variant is reported to increase the channel current of normal channels, while having lesser effects on the current of mutant channels [21]. Therefore, although its effect is not negligible, the p.Pro448Arg common variant does not strongly influence the JLNS/LQTS syndrome. Indeed, the son (III-1 in Fig. 2) of the proband was heterozygous both for p.E39X and p.P448R, which would have been inherited in cis from the proband. The son (III-1) has an equally convincing LQTS phenotype to his sister (III-2), who has inherited the two variants on one chromosome from her mother as well as the p.P448R common variant from her father.

Previous studies [23, 24] have reported a nonsense variant (NM_000218.2:c. 153 C > G, p.Tyr51X, in exon 1a) in a RWS patient. This patient appears to be the only other RWS patient harboring a nonsense variant in exon 1a; however, the patient has not been described in detail, besides her/his ethnicity being white [23, 24]. Although exon 1a is the kidney-isoform specific exon, the obvious QTc prolongation shown in our patient (III-1, III-2) leads to the reasonable conclusion that nonsense variants in exon 1a can cause RWS. Previous studies have reported several missense variants located in exon 1a (NM_000218.2:c.217C > A, p.Pro73Thr [23], c.1A > G, p.Met1Val and c.170G > T, p.Gly57Val [25] c.19C > T, p.Pro7Ser [26], c.136G > A, p.Ala46Thr [27]) that can cause RWS. A frameshift variant in exon 1a (NM_000218.2:c.151dupT, p.Tyr51Leufs*234) has also been reported [26, 27]. Although exon 1a is specific to the kidney isoform, variants in exon 1a can nonetheless cause RWS. According to the recent ACMG guidelines for the interpretation of sequence variants and pathogenicity [28], both the p.Glu39X and p.Tyr51X variants exhibit “very strong” evidence for pathogenicity.

Several papers have reported that families with JLNS exhibit variants affecting both KCNQ1 alleles, although family members harboring just one variant do not exhibit RWS symptoms or QTc prolongation [4, 12]. Conversely, it has been reported that some JLNS family members with heterozygous variant in KCNQ1, including our cases, show RWS symptoms or QTc prolongation [17]. In summary, some KCNQ1 variants can cause both JLNS (in an autosomal recessive manner) and RWS (in an autosomal dominant manner), while several KCNQ1 variants cause only JLNS in an autosomal recessive manner.

According to a comprehensive LQTs gene screening study [23, 24], the majority of RWS cases are caused by missense variants, rather than nonsense or frame-shift variants. In general, variants can cause disease by either haploinsufficiency or dominant-negative mechanisms. Several missense variants are reported to cause RWS by a dominant-negative mechanism [1]. It is reasonable to conclude that dominant-negative KCNQ1 variants can cause both RWS and JLNS.

An amino acid substitution in or nearby functional domains may cause loss of function or dominant negative effects similar to missense variants. The functional domains in the KCNQ1 gene are encoded in the middle (transmembrane domains) and downstream (subunit assembly domain) exons [23], which likely accounts for the majority of pathogenic variants being located in the middle and downstream exons.

On the other hand, nonsense variants in the region proximal to the N-terminus of exon 1a of KCNQ1 can cause significant QTc prolongations identical to RWS in our current and previous cases [23]. Nonsense variants in KCNQ1 are understood to be causative of haploinsufficiency for gene function [6]. Our case study supports that haploinsufficiency in the KCNQ1 gene is causative of significant QTc prolongation identical to RWS.

Furthermore, our case is the first demonstration of a variant in the kidney-isoform specific exon being causative of JLNS. Several genetically engineered mouse models of JLNS have been created, and two KCNQ1-knockout models have been described [29, 30]. Exon 1 (Fig. 1), the first common exon of the kidney and pancreas isoforms, is engineered in both knockout models. A JLNS family reported by Zehelein et al. [4] is similar to these models because the described variant is located in exon 1. Interestingly, significant QT prolongation is not observed in one model strain [29], while both deafness and QT prolongation are observed in the other KCNQ1-knockout model [30]. Our case is clearly a JLNS instance with both deafness and QT prolongation; however, corresponding animal models based on engineering of the kidney-isoform specific exon have not been developed.

As mentioned above, we have reported a unique variant of KCNQ1 (NM_000218.2:c.115G > T, p.Glu39X) that can cause not only JLNS, but also significant QTc prolongation identical to RWS. This nonsense variant may be informative to the genetic pathogenesis of RWS and JLNS caused by KCNQ1 variants. In the absence of corresponding animal models, iPS cell technology [31] has enabled the study of cell biology with gene variants. In the very near future, we aim to generate iPS cells with the KCNQ1 variant described herein.

## Additional file

Additional file 1: Table S1. The proband’s (II-2 in Fig. 2) results of genetic screening of LQT causative genes (KCNQ1, KCNH2, SCN5A, KCNE1, KCNE2, KCNJ2, SCN4B, KCNJ5). Entire coding exons, including the intronic boundaries of the genes were analyzed. (XLSX 10 kb)

## Abbreviations

DNA: Deoxyribonucleic acid
ECG: Baseline electrocardiogram
GRC: the Genome Reference Consortium
ICD: Implantable cardioverter defibrillator
iPS: Induced pluripotent stem cells
JLNS: Jervell and Lange-Nielsen Syndrome
KCNE1: Potassium voltage-gated channel subfamily E regulatory subunit 1
KCNE2: Potassium voltage-gated channel subfamily E regulatory subunit 2
KCNH2: Potassium voltage-gated channel subfamily H member 2
KCNJ2: Potassium voltage-gated channel subfamily J member 2
KCNJ5: Potassium voltage-gated channel subfamily J member 5
KCNQ1: Potassium voltage-gated channel subfamily Q member 1
LQT1: Long QT syndrome 1
LQTS: Long QT syndrome
mg: Milligram
ms: Milliseconds
msec: Milliseconds
NCBI: National Center for Biotechnology Information
ng: Nanogram
PCR: Polymerase chain reaction
QTc: Corrected QT
RWS: Romano-Ward syndrome
SCN4B: Sodium voltage-gated channel beta subunit 4
SCN5A: Sodium voltage-gated channel alpha subunit 5
